# Systematic Review on Resting-State EEG for Alzheimer's Disease Diagnosis and Progression Assessment

**DOI:** 10.1155/2018/5174815

**Published:** 2018-10-04

**Authors:** Raymundo Cassani, Mar Estarellas, Rodrigo San-Martin, Francisco J. Fraga, Tiago H. Falk

**Affiliations:** ^1^Institut national de la recherche scientifique (INRS-EMT), University of Québec, Montreal, Canada; ^2^Department of Bioengineering, Imperial College London, London, UK; ^3^Center for Mathematics, Computation and Cognition, Universidade Federal do ABC, São Bernardo do Campo, Brazil; ^4^Engineering, Modeling and Applied Social Sciences Center, Universidade Federal do ABC, São Bernardo do Campo, Brazil

## Abstract

Alzheimer's disease (AD) is a neurodegenerative disorder that accounts for nearly 70% of the more than 46 million dementia cases estimated worldwide. Although there is no cure for AD, early diagnosis and an accurate characterization of the disease progression can improve the quality of life of AD patients and their caregivers. Currently, AD diagnosis is carried out using standardized mental status examinations, which are commonly assisted by expensive neuroimaging scans and invasive laboratory tests, thus rendering the diagnosis time consuming and costly. Notwithstanding, over the last decade, electroencephalography (EEG) has emerged as a noninvasive alternative technique for the study of AD, competing with more expensive neuroimaging tools, such as MRI and PET. This paper reports on the results of a systematic review on the utilization of resting-state EEG signals for AD diagnosis and progression assessment. Recent journal articles obtained from four major bibliographic databases were analyzed. A total of 112 journal articles published from January 2010 to February 2018 were meticulously reviewed, and relevant aspects of these papers were compared across articles to provide a general overview of the research on this noninvasive AD diagnosis technique. Finally, recommendations for future studies with resting-state EEG were presented to improve and facilitate the knowledge transfer among research groups.

## 1. Introduction

The term dementia is used to characterize several neurodegenerative disorders caused by damage and death of neurons, provoking a disturbance of cognitive and behavioral functions. Among the different forms of dementia, Alzheimer's disease (AD) is the most common, accounting for nearly 70% of the dementia cases worldwide. It mostly affects people over 65 years of age and the rate of incidence grows exponentially with age [1–3]. Thus far, there is no cure for AD, only palliative treatments that temporarily slow the worsening of symptoms, aiming to improve the quality of life of patients and caregivers [4].

In 2015, 46 million people were diagnosed with dementia worldwide and this number is projected to grow to 66 million by 2030, and to 115 million by 2050 [3]. Given the aging population, much of the dementia cases (approximately 70%) will take place in low- and middle-income countries. Furthermore, dementia has significant social and economic impacts. For example, in 2015, the estimated worldwide cost of dementia was approximately 818 billion US dollars. By 2030, the financial burden is expected to increase to 2 trillion US dollars [3]. Due to evidences on the global prevalence and incidence of dementia, associated mortality, and global economic cost, the World Health Organization made an urgent call to include dementia as a priority in health agendas around the globe in order to raise awareness, improve early diagnosis, and provide better care and support to patients, families, and caregivers [1]. Moreover, unlike other health problems which have reported declining incidence over recent years, the number of deaths caused by AD has increased by 89% between 2000 and 2014 [3].

According to symptomatology, AD has been divided in three stages: preclinical, mild cognitive impairment, and dementia due to AD [5].

*(1) Preclinical AD.* Changes in the brain, blood, and cerebrospinal fluid related to AD start to occur, but the patient does not show any symptoms. This phase may start up to years or decades before the first clinical symptoms of dementia [6, 7]. The possibility of detecting AD in this preclinical stage would offer a pivotal opportunity for therapeutic interventions [8].

*(2) Mild Cognitive Impairment (MCI).* In this early stage, the person still functions independently but may feel some memory lapses and difficulties coming up with the right word or remembering the location of familial places. Friends and family may notice these small difficulties. This stage is often referred as mild or early-stage AD disease. The term MCI has been frequently used in research trials with the objective of including as many individuals as possible with symptoms that were not severe enough to meet current AD diagnostic criteria but might at some point in time. However, it has been observed that 30% of subjects diagnosed as MCI will not progress to AD dementia in a near future [9–11].

*(3) Dementia due to AD.* The patient's ability to function in daily life is seen affected by impairments in memory, thinking, and behavior [12]. This stage is frequently subdivided into the following:

*(a) Moderate or Middle-Stage AD.* In this phase, usually the longest one, the person may experience greater difficulty to execute daily tasks such as paying bills, recalling own address, getting dressed, or controlling bladder and bowels. The patient notices these symptoms, which leads to frustration and anger. Also, in this stage, some psychological symptoms start to appear, e.g., suspiciousness, delusions, or compulsive behavior.

*(b) Severe or Late-Stage AD.* In this final stage, individuals start losing their ability to interact with the environment and their memory and cognitive skills are severely affected. In this phase, the patient needs 24-hour personal care.

The pathophysiological process of AD is thought to start up to 20 years before clinical symptoms can be detectable [6, 8]. In the last two decades, evidence has shown that the correspondence between pathology and clinical symptoms is not always consistent [5]. Indeed, the pathology and clinical symptoms in AD are best conceptualized as separated continua, which may evolve in parallel but with a temporally offset. [6]. As such, nowadays, AD is regarded as continuum rather than discrete stages [7, 13].

Accurate diagnosis is a true challenge, as AD pathophysiological processes may start up to 20 years before clinical symptoms can be detectable [8, 10]. Also, AD symptoms are commonly confused with normal aging processes, thus frequently delaying diagnosis [9]. Being able to diagnose AD in its early stage would give the patients and their families time to prepare themselves emotionally and financially for the years to come. An accurate early diagnosis would also help patients to preserve their independence longer and prevent psychiatric-related symptoms such as depression or psychosis, thus reducing personal and societal costs associated with AD [14]. Moreover, it is likely that the effectiveness of novel drugs for AD symptom treatment will be higher in early stages of the disease, before neurodegeneration is irreversible or too extended [15].

Today, definite AD diagnosis is only possible postmortem when analysis reveals the structural brain damage characteristic of the disease. Typically, accuracies up to 90% have been reported with current diagnosis methods, such as neurological tests and medical records. The current clinical diagnostic criteria for AD were developed by the National Institute on Aging and the Alzheimer's Association (NIA-AA) [5, 6, 11, 12]. These criteria are an update of the previous widely used guidelines established in 1984 by the National Institute of Neurological and Communicative Disorders and Stroke and the Alzheimer's Disease and Related Disorders Association (NINDS-ADRDA) [16]. These updated guidelines include the use of neuroimaging and cerebrospinal fluid (CSF) biomarkers to support a diagnosis of AD in symptomatic individuals [5]. Additionally, the European Federation of the Neurological Societies (EFNS) also developed a guideline to diagnose and monitor AD [17]. The most used test to measure cognitive ability for AD diagnosis is the Mini Mental State Examination (MMSE) [18, 19]. The Montreal Cognitive Assessment (MoCA) [20] and Addenbrooke's Cognitive Examination revised (ACE-R) [21] are also frequently used in clinical practice. Other examples of neurological tests are the Severe Cognitive Impairment Scale, the Alzheimer's Disease Assessment Scale Cognitive, the neuropsychological test battery, and the Severe Impairment Battery [9]. Moreover, the Trail Making Test (TMT) [22] and the clock drawing test [23] focus not only on measuring cognitive abilities but also on attention and executive functions. The Rey Auditory Verbal Learning Test and the category fluency test, in turn, also measure patient construction praxis ability [24]. Additionally, other disorders that also lead to dementia as vascular brain injury, Lewy body diseases, and Parkinson disease in some cases are also comorbid to AD [25]. The differential diagnosis between AD and these disorders in their early stages is strengthened by the usage of techniques that access specific biomarkers as some early symptoms overlap [26].

Relying on neurological tests and the evaluation of medical records require experienced clinicians and lengthy sessions, rendering AD diagnosis irreproducible and time consuming. In response to these drawbacks, in the last few years, there has been an increase in the use, research, and development of biomarkers [9]. These biomarkers play a central role in the recent research diagnosis criteria for AD [9, 13, 27]. Biomarkers can be divided in three main categories: A, T, and N, where the first two categories include biomarkers that measure the brain amyloidosis and tauopathy, respectively, e.g., amyloid and tau tracer PET (positron emission tomography) scans, and CSF concentrations of A*β*142 and P-tau and category N encompasses biomarkers that measure neurodegeneration or neural injury (e.g., CFS T-tau, FDG PET, and atrophy in MRI) [13]. It has been found that *Aβ*42, the most common CSF biomarker, presents lower values in AD patients compared to healthy individuals [28]. However, to obtain a CSF sample, a lumbar puncture is required, making this technique invasive, thus hindering its use in daily clinical practice. As an alternative, blood biomarkers such as plasma T-tau are also in the search, as they can provide similar information as CSF but are a less invasive and expensive technique [29]. Neuroimaging tools such as magnetic resonance imaging (MRI), computed tomography (CT), and PET allow clinicians the investigation of brain damage extension due to AD in vivo. However, once the disease-related structural damage is detectable by the current spatial resolution of these neuroimaging techniques, AD is already well advanced, i.e., the atrophy in the brain is already extended [9]. Moreover, these neuroimaging tools are expensive and time consuming and require intervention by experts. Also, not all hospitals can afford MRI and PET scanners, particularly in low- and middle-income countries or remote regions, thus leading to displacements that are neither comfortable nor practical for the patient. Unfortunately, wide utilization of existing CSF-derived biomarkers and neuroimaging techniques is not practical, as these techniques are either invasive or costly. Therefore, an alternative or supporting technique that allows easier and more convenient AD diagnoses is needed. This is where electroencephalography- (EEG-) based biomarkers have come in.

EEG is a technique that consists of recording the changes in time of the electrical activity in the cerebral cortex, produced by postsynaptic potentials from thousands of neurons with similar spatial orientation. These electric potentials are measured by electrodes placed on the scalp. The spatial resolution of EEG is related to the number of electrodes used and their placement, or layout, on the scalp. The most utilized layout is the international 10-20 system, commonly consisting of 21 electrodes; higher density variants of the 10-20 system such as 10-10 and 10-5 systems are utilized as well, usually with 64 and 128 electrodes, respectively, [30] as well as the alternative layouts Maudsley [31] and Geodesics positioning systems [32]. In recent years, quantitative EEG (qEEG, henceforth simply EEG) has been proven to be a reliable clinical tool for the diagnosis and study of illnesses and cortical disorders such as Huntington disease [33], autism spectrum disorders [34], epilepsy and seizure [35], cerebral ischemia [36], frontotemporal dementia [37], and Parkinson's disease dementia [38]. Furthermore, the differential diagnosis between AD and other diseases that lead to dementia as vascular brain injury [39, 40] and Lewy body diseases [41, 42] was assessed with EEG. In the analysis, EEG signals are commonly divided into 5 major frequency bands, namely, delta (*δ*) 0.1–4 Hz, theta (*θ*) 4–8 Hz, alpha (*α*) 8–12 Hz, beta (*β*) 12–30 Hz, and gamma (*γ*) *>* 30 Hz. Moreover, further divisions in these bands are considered (low alpha, high alpha, low beta, etc); however, the frequency limits for the subbands are not standardized across studies. Each frequency band conveys different information about brain functionality and synchronization [43–45].

Since EEG signals reflect functional changes in the cerebral cortex, EEG-based biomarkers can be used to assess neuronal degeneration caused by AD progression (biomarkers in category N according to [13]), long before actual tissue loss or behavioral symptoms appear. As such, EEG is a promising technique with the potential of serving as support and/or alternative to existing tools (e.g., CSF and MRI/PET), but with the advantage of being noninvasive, portable, and less expensive. Furthermore, EEG has better temporal resolution than other neuroimaging techniques [45, 46]. EEG signals have been studied in healthy elderly people, showing that there are no substantial changes in EEG associated to healthy aging, making EEG a suitable technique for AD and other dementia assessment [47]. One of the major shortcomings of the EEG signal lies in its sensitivity to signal artifacts, such as eye blinks and movements, heartbeats, cranial muscle activity, and power grid interference. These artifacts have detrimental effects on EEG signal quality, reducing the AD diagnosis performance.

### 1.1. EEG Recording Conditions and Reported AD Effects

Over the last decades, many studies have investigated the effects of AD and its progression on EEG signals. Studies have made use of EEG signals under diverse recording conditions, which can be divided into two major groups:

*(1) Resting-State EEG Recordings.* Spontaneous EEG activity is recorded during the absence of any kind of stimulus, thus measuring the brain background activity. As the participant is not required to perform any specific task, EEG acquisition becomes simpler, more comfortable, and less stressing for the patient, especially for elderly individuals [48]. Resting-state EEG recordings comprehend recordings in the resting-awake state (either open or closed eyes) and recording during sleep. Four typical effects of AD on resting-state EEG signals have been repeatedly observed:

*(a) Slowing.* Power spectrum shifts from high-frequency components (alpha, beta, and gamma) towards low-frequency components (delta and theta) have been commonly seen in AD patients [14, 49, 50]. This shift is proportional to the progression of AD and is thought to be the result of loss of cholinergic innervations in AD patients. Features derived from the power spectrum, power spectrogram, and wavelet analysis have been used to quantify this slowing of the EEG.

*(b) Reduced Complexity.* A decrease in the complexity of the brain electrical activity has been observed in AD patients compared with healthy controls [14, 50–52]. This decrease is likely caused by massive neuronal death and reduced connections in cortical regions, leading to simpler EEG dynamics. Some signal processing techniques employed to study the complexity of EEG signals are entropy measures, automutual information, Lempel-Ziv complexity, fractal dimension, and Lyapunov exponent.

*(c) Decrease in Synchronization.* Manifested as a reduction in connectivity between cortical regions, this has been seen in many AD patients. The cause behind this phenomenon is not well understood, although it is thought to be related to the atrophy in the communication of neural networks [53–56]. The techniques used to study this effect are Pearson correlation coefficient, magnitude coherence, phase coherence, Granger causality, phase synchrony, global field synchrony, and cross-frequency coupling. It is interesting to mention that some studies, contrary to the majority, have shown an increase of synchrony in patients with MCI and AD, which is thought to be caused by compensatory mechanisms in the brain [57].

*(d) Neuromodulatory Deficits.* Amplitude modulation analysis has recently been proposed to quantify EEG rhythms and the neuromodulatory activity of the brain via cross-frequency interaction effects [58].

*(2) Event-Related EEG Recordings.* EEG signals are recorded in relation to the occurrence of a specific event, i.e., signals are time locked. Besides being time locked, EEG activity is also phase locked and thus receives the name of event-related potentials (ERP). When the EEG activity is not phase locked, it is called induced activity [59, 60] and can be analyzed either by event-related (de) synchronization (ERD/ERS) [44, 61] or by event-related oscillations (ERO) [62]. Events can be related to sensorial perceptive, motor, and cognitive processes [43, 45, 62].

In the literature on AD, recent reviews have been published covering the use of event-related EEG for AD diagnosis [62–64]. While event-related EEG recordings offer the opportunity to examine the effect of AD on specific brain circuits, these recording conditions are not ideal for most AD patients, since even from early AD stages, people experiment an increase in anxiety and anger, as well as a decrease in the desire in having new experiences. Therefore, even the performance of a simple memory task might cause discomfort and anxiety to the patient; they might feel disoriented or unable to complete it [65–68]. On the other hand, resting-state protocols do not require external stimuli and thus they are simpler and more comfortable for the patients. Moreover, such protocols also have fewer artifacts.

Regarding resting-state analysis for AD diagnosis, some recent reviews have also been written. However, none of these have treated exclusively the specific topic of EEG-based AD diagnosis. For instance, some reviews do not study EEG as the main technique for diagnosis [9, 47, 69–74], while others are exclusively focused on the synchronization of EEG signals [55, 56]. Moreover, other publications provide a broader review of the whole dementia spectrum and not only AD [24, 46, 70, 71, 75]. Main feature categories for AD diagnosis are extensively discussed in revisions [14, 50, 57]. As such, the present study complements previous EEG-based AD diagnosis reviews by systematically and exclusively reviewing articles on resting-state EEG, to provide a systematic overview of the current state of the art.

### 1.2. Aim of the Review

This systematic review will focus on recent studies on resting-state EEG for AD diagnosis, describing and comparing the crucial stages in EEG-based AD diagnosis, such as EEG signal acquisition, preprocessing, artifact handling, and feature extraction and classification. Moreover, pointing out common practices, differences and consensus in the utilization of resting-state EEG reported limitations and recommendations for several experimental stages ranging from population characteristics to results reporting for future studies. We hope that this review will boost the research on this topic, leading to more reliable EEG resting-state AD diagnosis techniques. The remainder of this article is organized as follows.  Section 2 describes the methods and steps carried out in this systematic review.  Section 3 presents and discusses the review results, with a list of recommendations for future EEG-based AD diagnosis studies. Finally, the conclusions are presented in Section 4.

## 2. Methods

A survey on English peer-reviewed journal articles published between January 2010 and February 2018 was performed for this review. Four major bibliographic databases were queried, namely, PubMed, Web of Science, IEEE Xplore, and Scopus, using the following search terms:

1. ^∗^EEG
2. Electroencephalogr^∗^
3. Alzheimer^∗^
4. Diagnos^∗^

These search terms were combined in the following rule: (1 OR 2) AND 3 AND 4. Resulting journal articles were selected or rejected based on the criteria presented in Table 1.

Along with the mentioned search terms, studies that used other modalities besides EEG were further analyzed and divided in two types: (i) those that used features from other modalities combined with EEG features for AD diagnosis and (ii) those that used other modalities to verify and compare the results obtained with only EEG. The former papers were excluded, as we want to focus only on EEG-based diagnosis of AD; the latter were included.

Eligibility assessment was performed by at least two independent researchers by reading the article title and, when the article title did not provide enough information to be selected or rejected, the abstract was also read. In the cases where the assessors independently disagreed on the inclusion or exclusion of a paper, the final decision was made after a discussion between the two. Lastly, some articles were rejected after careful reading of the papers when it became clear that it did not meet the inclusion criteria. In order to keep track of the relevant information while reading the articles, a data extraction sheet was developed. For each article selected, 21 data items were extracted and grouped into five categories: study rationale, study population, experiment setup, EEG processing, and reported outcomes.

The category *study rationale* includes article elements that are related to the study aim, studied groups, and combination of other types of dementia when it applies. The second category, *study population*, focuses on elements related to subjects, such as the number of participants and whether the cohorts have been matched by sample size, age, gender, and education level. In the category *experiment setup*, in turn, items are associated to the reported experiment protocol, number of electrodes used, and extra bioelectrical signals acquired simultaneously, as well as recording conditions and experiment duration. The *EEG processing* category includes preprocessing techniques, bandwidth of EEG signals, artifact rejection and/or correction methods, frequency sampling used for feature extraction, EEG epoching, source localization when used, and feature types. The last category, *reported outcomes*, gathers the reported results with different study goals/protocols and the reported limitations. These five categories and their respective subitems are described in Table 2.

This review was written following the PRISMA statement scheme for reporting systematic reviews [76].

## 3. Results and Discussion

**Figure 1:**
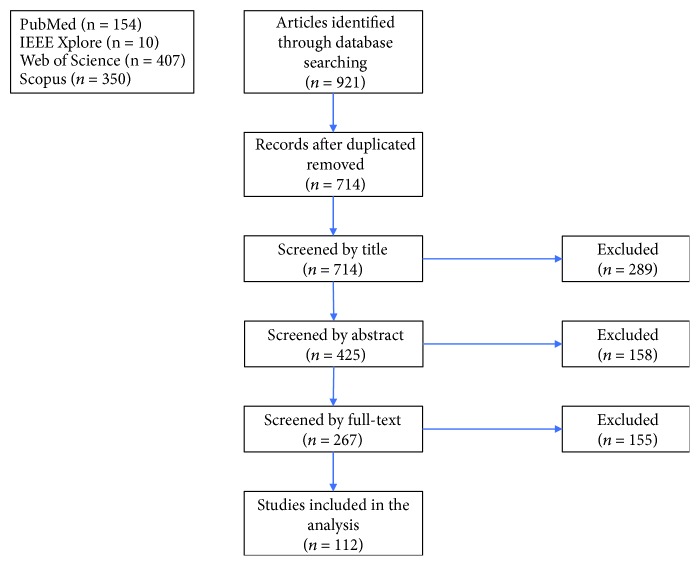
Diagram showing the selection process of articles from PubMed, IEEE Xplore, Web of Science, and Scopus.

**Figure 2:**
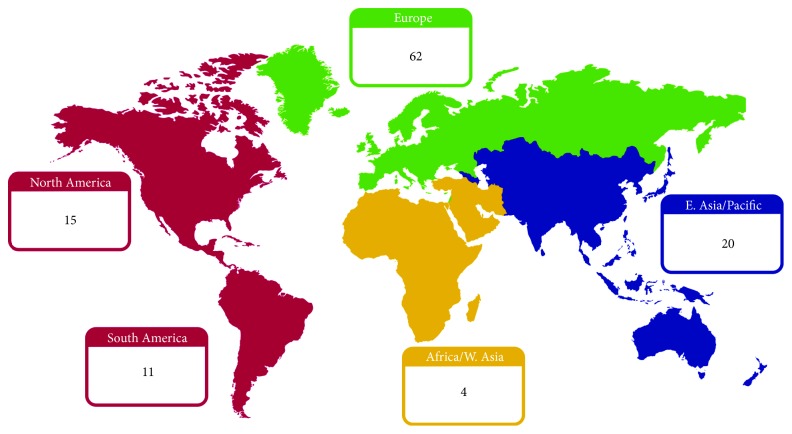
Distribution of selected articles according to world regions.

**Figure 3:**
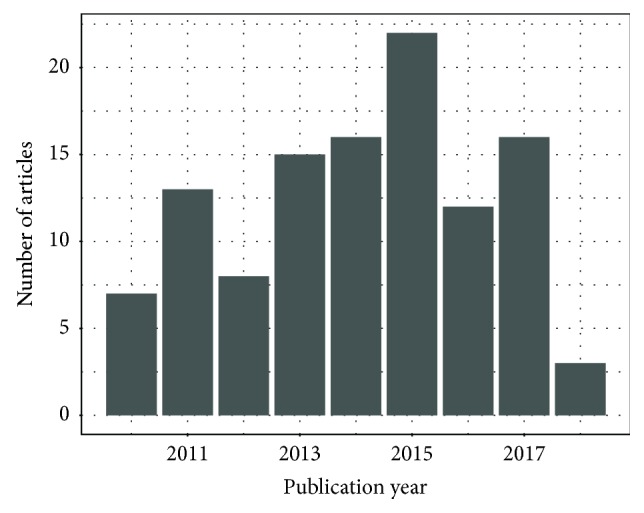
Number of reviewed articles by publication year.

A total of 921 journal articles were found in the database queries, with 714 unique records remaining after duplicates were removed. Through title and abstract screening, 289 and 158 articles were rejected respectively, as they did not meet the inclusion criteria. A total of 267 articles met all the previously established inclusion criteria. After full-text examination, only 112 articles were included in the systematic review.  Figure 1 depicts the abovementioned selection process. The geographic distribution of the papers, according to first author institution, is detailed in Figure 2. The temporal distribution of articles published between 2010 and 2018 is shown in Figure 3. The items defined in Table 2 were extracted from each article, and the following subsections present a direct comparison on these items across the reviewed articles.

### 3.1. Study Rationale

#### 3.1.1. Study Goal

According to the reported aim of the articles, two major goals were identified: (1) discriminative (or diagnosis), i.e., explore the difference in EEG-based features among populations, MCI, mild AD, severe AD, other types of dementias, and healthy normal elderly controls (Nold) and (2) progression assessment, i.e., find correlates between EEG-based features and clinical markers related to the MCI-to-AD conversion and AD severity progression. The majority (72) fell exclusively in the diagnosis category, whereas 18 articles were included in the progression assessment category and 22 studies were double aimed. The articles belonging to each study goal and populations investigated are presented in Table 3.

#### 3.1.2. Combinations with Other Dementias

As mentioned previously, dementia is a term that involves different disorders and diseases, one of them is AD, which accounts for the great majority of dementia cases. Having similar symptoms, around 10% of dementia cases are difficult to diagnose with reasonable confidence and it is not uncommon in clinical practice to mix dementia diagnoses [130]. As such, from the reviewed studies, ten studied the potential of EEG-based features to perform a differential diagnosis among types of dementia. In Table 4, a list of other dementia types explored by those studies is presented. In those studies, distinctions in spectral slowing features between AD and other dementias are identified for vascular dementia [26, 39, 130], frontotemporal dementia (FTD) [37, 130, 175, 176], Lewy body dementia (DLB) [130, 176], and Parkison's disease dementia (PDD) [130]. Moreover, disparity in synchronization measures is reported between AD, PDD [38, 42], DLB and FTD [42]. Additionally, a combined model of EEG and MRI improved discrimination between AD and DLB [41].

### 3.2. Population Characteristics

#### 3.2.1. Number of Subjects

**Figure 4:**
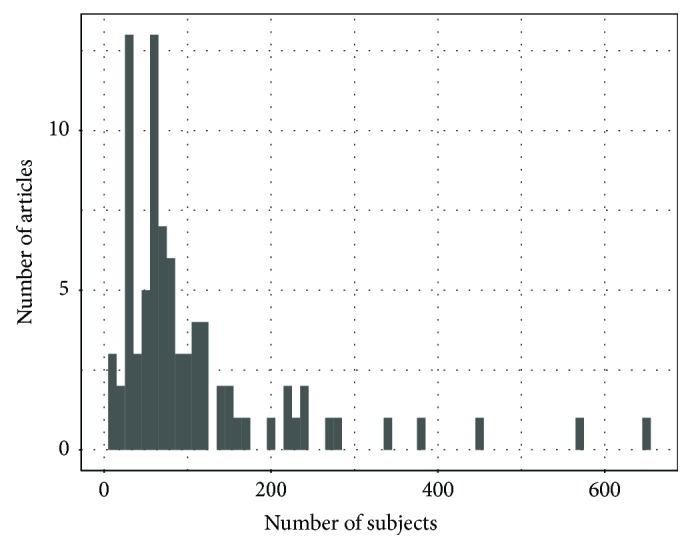
Number of subject histogram.

The number of participants reported in each paper varied greatly, ranging from 12 to 654 subjects as shown in Figure 4. From the total 112 articles, 84 unique datasets were utilized, since 11 were used by more than one study, as presented in Table 5. Dataset diversity is desirable to avoid biases and overfitting in the models. Unfortunately, there is also a great diversity in the dataset acquisition variables such as electrode montage, number of electrodes, sampling frequency, and EEG recording conditions. These differences among datasets make it difficult and sometimes even impossible to evaluate developed methods across different datasets.

#### 3.2.2. Group, Age, Education, and Gender Matching

In most studies, the number of participants per group is well balanced between healthy controls, AD patients and, in some cases, MCI patients. Notwithstanding, 15 studies only included one group and this was the case of most studies in the progression category (Table 3). As detailed in Table 6, 56 from a total of 97 studies exploring the difference between two populations or more are balanced in relation to the number of subjects. Age, education, and gender are also possible confounding factors that influence AD diagnosis [145, 162]. From the 97 studies that included a healthy control group, 65 matched groups for age, 27 for gender, and 25 for years of education. In total, only 8 studies [26, 37, 66, 81, 103, 115, 124, 176] paired groups for number of subjects, age, education, and gender. Some studies did not inform group matching for some of the variables; thus, they were considered as not paired for these variables.

#### 3.2.3. Following of MCI

Forty studies included MCI participants, as detailed in Table 3. The aim of these studies was to investigate EEG-based biomarkers to discriminate or characterize early AD. Among these, only thirteen [124, 125, 127, 134, 135, 147–149, 151, 159, 177–179] reported follow-up information on these patients. AD conversion rate in MCI stands between 70 to 80%, whereas the rest of these patients can continue stable or convert to other dementias [181]. In this way, assuming MCI condition as a prodromal, AD stage might introduce bias in reported results. On the other hand, MCI patients who converted to AD can be considered as early AD. Thus, longitudinal studies are recommended as they can provide a more homogeneous group classification.

### 3.3. Experimental Setup

#### 3.3.1. Combination with Other Modalities

When EEG recordings were utilized along with other techniques such as MRI, PET, and CSF analyses, only studies that reported only-EEG-based diagnosis or assessment were considered. A total of 92 articles used exclusively EEG for their studies. Nevertheless, as other biomarkers have been explored and validated by the clinical community (e.g., CSF and MRIs), comparisons between these modalities and EEGs are very useful.  Table 7 shows other modalities combined with EEG and the biomarkers derived from them.

#### 3.3.2. Number of EEG Electrodes and Layout

The reported number of electrodes used for EEG signal acquisition in the reviewed studies varies greatly, from as low as one to as high as 256 electrodes (Table 8), being 19 electrodes the most common number (53 studies). The decision regarding the number of electrodes is driven by the trade-off between spatial resolution and participant comfort. EEG systems with 32 or more channels are cumbersome and its electrode placement/adjustment can take around one hour or even longer. Long pretest procedures may provoke drowsiness, fatigue, stress, and/or alternate mental states that may alter EEG patterns and, consequently, study outcomes [85]. Another point to be considered, detailed in subsequent Section 3.4.7, is the minimal density required in source location analysis, as greater numbers of channels increase precision [182]. Regarding electrode layout, 107 studies used the 10-20 international positioning system (and its variations, 10-10 or 10-5 systems) or the Maudsley system; the remaining five studies [77, 98, 99, 124, 128] acquired EEG signals with 110 or more electrodes using the geodesic system.

#### 3.3.3. Additional Channels

During EEG recordings, it is common practice to acquire simultaneously electrooculogram (EOG) and electrocardiogram (ECG) signals to monitor eye movement and heart activity, respectively. EOG and ECG are helpful as reference for cleaning the EEG signals as ocular and heart activity artifacts will be easier to detect and clean. Forty-one studies mention the registration of EOG signals in their studies [42, 66, 81, 82, 89, 103, 115, 130, 131, 133, 136–139, 141, 143–147, 150, 152–160, 162, 164–166, 169, 170, 172, 174–177] and twelve mention the use of ECG [38, 42, 131, 144–146, 161–163, 168, 172, 175].

#### 3.3.4. Resting-State Recording Conditions

Resting-state EEG can be recorded under two different conditions: sleeping and resting awake (either open or closed eyes). From the reviewed articles, the most common recording condition was resting awake eyes closed (EC), reported in 109 studies. None of the reviewed articles acquired EEG during sleep, or solely during resting awake eyes open (EO). Taking into account that the vast majority of participants are elderly and around half of them suffer from AD or MCI, resting-awake conditions are the most comfortable recording condition for participants, as they are not required to perform any mental task, which could be confusing or frustrating for these individuals [56]. Moreover, resting-awake conditions reduce artifacts due to head movement- and in the most common case of EC also eye movement-related artifacts. Conversely, there are some studies that hypothesize that recording EEG under certain tasks may lead to higher discrimination power, since those tasks can be designed to probe specific brain regions and pathways affected by AD [14, 91]. In addition to resting-state recordings, 11 studies also reported EEG recorded during sensory stimulation or cognitive tasks.  Table 9 presents the EEG recording conditions utilized in each reviewed article.

#### 3.3.5. Experiment/Signal Duration

The total duration of the EEG recording session was reported in 82 articles. This is important as long sessions can have detrimental effects for wet electrodes and cause alterations in participant mood and compliance [183, 184]. The reported recording times varied from two up to 33 minutes, with 10 minutes being the average duration.  Table 10 summarizes the EEG session length when reported.

### 3.4. EEG Signal Processing

#### 3.4.1. Preprocessing

In a broad sense, EEG signal preprocessing stands for the manipulations performed on the raw acquired data in order to prepare it for feature extraction in the next processing phases [43–45]. Most of these techniques are common to almost all neuroscience EEG studies, not only to AD diagnosis. With preprocessing techniques, desired spectral components of the acquired EEG signals are enhanced and noise is removed; this is typically performed with digital filters used in the time domain. The most common preprocessing techniques include band-stop or notch filters to remove power grid interference (50 or 60 Hz, depending on the country), bandpass filtering to enhance only EEG-related spectral components, resampling, EEG rereferencing, and bad channel rejection or interpolation.  Table 11 shows the reported preprocessing techniques used in the reviewed articles.

#### 3.4.2. EEG Bandwidth

The most common approach is the use of digital bandpass filters to enhance EEG-related spectral components. As each study had specific interest in different spectral components, diverse bandwidths have been reported. Lower bound of the EEG bandwidth is usually in the range of 0.1 to 4 Hz; however, the upper bound varies in a wider range, from 20 to 200 Hz. The most common upper limit was 70 Hz (31 studies) and the most used lower limit was 0.5 Hz (36 studies). Tables 12 and 13 present, respectively, the different upper- and lower-frequency bounds used in the reviewed articles.

Moreover, filtering can be performed with finite impulse response (FIR) or infinite impulse response (IIR) filters. The types of filters used in the various studies are presented in Table 14. This is important, as the use of IIR filters may distort the signal due to phase nonlinearity, therefore critical for studies analyzing connectivity based on phase.

#### 3.4.3. Artifact Handling

EEG signals are inherently noisy and susceptible to blink, eye movements, heartbeats, cranial muscle, and power line artifacts. As mentioned previously, the process of cleaning EEG data from artifacts is pivotal in the EEG signal preprocessing pipeline. Analyzing clean EEG signals is an important prerequisite to avoid errors in the feature extraction step and to prevent misclassification of mental activity [84]. To overcome the detrimental effect of artifacts, the majority of the reviewed studies (65) reported the use of artifact-free EEG segments manually selected by expert clinicians through meticulous visual inspection. This is a time-consuming, expensive, and prone to human error process. Nine papers reported the use of semiautomated methods based on the ICA (independent component analysis) method, which also require human intervention to label components as artifactual. Lastly, 18 articles made use of automated artifact removal (AAR) methods, such as FASTER and wavelet-enhanced independent component analysis (wICA), which are able to substitute the human intervention in the artifactual component selection [85, 124], or of linear regression on electromyographic electrodes or of a notch filter tuned to the blink frequency [138]. In [84], different AAR algorithms were compared to evaluate their impact in the AD classification performance compared to raw and manually selected EEG signals and wICA was found to give the best results.  Table 15 shows the artifact handling approaches reported in the reviewed articles.

#### 3.4.4. Effective Sampling Frequency

While EEG devices can digitize data at high sampling frequencies (in the order of kHz), EEG signals are often downsampled as the processing of signals with excessive temporal resolution results in extra (and perhaps not as useful) computation load. As such, Table 16 lists the sampling frequency at which the EEG signals were processed, i.e., the effective sampling frequency, for the reviewed articles.

#### 3.4.5. EEG Epoching

The EEG signal is not stationary; however, it presents quasi-stationarity behavior for epochs (segments) ranging approximately from 1 to 60 s [154]. From the reviewed articles, the most common epoch duration was 2 s, used in 26 studies. The reported epoch lengths are presented in Table 17.

The EEG epoch length is quite consistent across studies, with 56 studies using 5-second epochs or less. On the other hand, the number of epochs used in EEG analysis varies greatly from study to study. The utilization of overlapping epochs to extract EEG features and their averaging in the feature domain has been shown to improve the features of SNR and, consequently, increasing the classification performance [185].  Table 18 presents the number of epochs reported in the reviewed articles.

#### 3.4.6. Effective EEG Signal Length

Study used several EEG epoching approaches (Section 3.4.5); thus, a direct comparison is not possible. For this reason, we proposed the effective EEG signal length as a metric to allow comparison among studies. The effective EEG signal length is given by the epoch length multiplied by the number of epochs used. This metric indicates how much data from the originally acquired EEG is kept for further processing.  Table 19 presents the values of this effective EEG signal length.

#### 3.4.7. Source Localization

EEG source localization methods estimate the location and distribution of active (electric) current sources within the brain based on the potential recorded through scalp electrodes. Going from activity recorded with electrodes to the current sources is an ill-posed inverse problem, since the number of unknown parameters is greater than the number of known parameters. In the last decades, this has been proven useful as a noninvasive neuroimaging technique, with high temporal and low spatial resolution that allows the characterization of “inside-the-brain” activity. A review on EEG source localization can be found in [186]. Among the reviewed articles, 17 studies used source localization methods for characterizing AD. Fifteen of these articles utilized the low-resolution electromagnetic tomography (LORETA) method or its derivatives (eLORETA and sLORETA) [26, 37, 66, 77, 81, 82, 103, 115, 128, 131, 141, 150, 163, 169, 176]. The remaining two articles used the local autoregressive average (LAURA) source localization method [99, 171]. Five papers using source localization methods [66, 77, 99, 128, 171] used medium- to high-density electrode montages ranging from 64 to 214 electrodes; the 12 remaining used 19 electrodes. A higher number of electrodes improve source localization precision, with a ceiling effect at 100 electrodes [182]. Nevertheless, as mentioned in Section 3.3.2, the comfort of the patient should be taken into account in the experiment design.

#### 3.4.8. EEG Features

As already reported in Section 1.1, four major effects of AD in resting-state EEG signals have been reported in literature. Most of the studies reviewed herein proposed and used one or more types of EEG features for AD characterization. As such, reported EEG features were grouped into five categories; the first four categories encompass features which aim at measuring one major effect of AD in the EEG signal (slowing, complexity reduction, synchronization decrement, and neuromodulatory deficit) and the last category termed “other” includes data-driven features which are not necessarily driven by known biological processes. It was frequent to observe the fusion of features from multiple categories in the same article. The description of each EEG feature herein presented is beyond the scope of this review; the interested reader is referred to the corresponding articles listed in this section for the feature definitions.

*(1) Slowing of the EEG Signals.* The measurement of the slowing effect on EEG signals due to AD typically relays on spectral features derived either from each of the EEG channels or from the average across channels. Alternatively, the estimated current source densities (obtained with source localization) can be analyzed for each frequency band. The reported slowing features are subdivided into three categories: current source density, spectral, and spectrotemporal (Table 20).

*(2) Reduction in Complexity in the EEG Signals.* Complexity of EEG signals is typically evaluated with entropy measures. Techniques used for this evaluation vary greatly among the reviewed articles, as detailed in Table 21.

*(3) Decrease in Synchronization.* The different metrics to measure the synchronization of EEG signals can be divided according two criteria: (1) the presence or absence of directionality (causation) information and (2) if the metric assumes a linear relationship between the analyzed signals (model based) or no assumption of linear relationship (model free) [187]. As such, reported synchrony features are divided into five categories: nondirected model based, nondirected model free, directed model based, directed model free, and others (Table 22).

*(4) Neuromodulatory Deficits in EEG Rhythms.* These changes have been explored by analyzing the statistics or the spectral content on the amplitude modulations for each of the classical EEG frequency bands. The spectral analysis of the amplitude modulations were proposed according to AD treatment literature, which suggested that neuromodulatory deficits seen with AD could be treated via deep brain stimulation, since according to the hemoneural hypothesis, cerebral hemodynamics might play an important role in information processing through the modulation of neural activity [58]. Reported neuromodulatory features are presented in Table 23.

*(5) Other Data-Driven Features.* Some studies used data-driven methods to derive features to differentiate dementia patients. These features do not have a clear relationship with known biological effects on EEG. These articles were classified in Table 24.

### 3.5. Reported Outcomes

As reported in the study goal (Section 3.1.1), results herein are analyzed according to their objective: discriminative or assessment/progressive.

#### 3.5.1. Discriminative Studies

The reported findings for discriminative studies fall into three categories: (1) studies reporting statistical significance of used features, (2) studies reporting classification performance among populations, and (3) studies reporting both statistical significance and classification performance.  Table 25 presents the discriminative studies according to these categories.

Papers where statistical significance was reported used a variety of parametric and nonparametric methods for statistical analysis.  Table 26 presents the statistical tests utilized in the reviewed articles. Normally a *p* value ≤ 0.05 was considered statistically significant and in some cases only when *p* ≤ 0.01. Analysis of variance (ANOVA) is also commonly used to find differences in extracted features between groups.

In studies where classification performance was reported, three important aspects were taken into account: feature selection, cross-validation, and classification algorithm.

*(1) Feature Selection.* The use of high-dimensionality feature vectors on limited data (few feature vectors per subject) often leads to bias and overfitting in classification. Moreover, many features might be correlated and do not provide new information to the classification algorithms and thus need to be removed. For this purpose, several feature selection methods have been proposed in the literature (Table 27). From the 59 articles where classification is performed, 27 reported the use of a feature selection method. In Table 27, a compilation of the reported feature selection methods is presented, being the area under the curve (AUC), *p* value, and support vector machine (SVM) the most used methods.

*(2) Cross-Validation.* In the process of training and testing classification algorithms, usually, the dataset is split in two in order to perform each task, as a measure to avoid optimistic bias and improve generalization. This data partitioning is called cross-validation (CV) [189]. From the 59 articles performing classification, leave-one-subject-out (LOSO) was the most used CV paradigm, employed in 24 studies. Under this paradigm, in a dataset with N participants, data from N-1 subjects is used to train the classifier, while data from the remaining subject is used for testing. This procedure is repeated N times, such that all subjects have their data become the test set once.  Table 28 presents the reported cross-validation paradigms.

*(3) Classification Algorithms.* The classification process refers to the assignment of discrete labels to a feature vector. Thus, the role of a classifier algorithm is to learn from data the transfer function between feature vectors and labels [189]. The reported classification algorithms are presented in Table 29, being the support vector machine (SVM) algorithm the most commonly used. Classification accuracy was widely used as the performance metric. However, given the discrepancies in experiment setup, EEG processing pipeline, and cross-validation paradigms, there is no way to directly compare the results.

#### 3.5.2. Assessment/Progression Studies

A total of 39 studies aimed to find correlates between EEG-based features and AD progression.  Table 30 presents a list of the methods utilized to evaluate the usability of the proposed EEG features. Pearson correlation was the most used method in order to see relationships between the EEG features and either mental examination scores (MMSE) or varying neuroimaging biomarkers, such as cortical thinning, brain perfusion, and other MRI/PET features.

#### 3.5.3. Reported Limitations

By compiling the different limitations reported in all the reviewed articles, it is possible to have an idea of the issues that need to be addressed in the following years to advance EEG-based research on AD. Firstly, the most reported limitations are related to the population participating in the studies, specifically, the small size of the dataset and cohorts (Section 3.2.1); the difficulty in age, gender, and/or education matching (Section 3.2.2); and AD participants taking antidementia drugs. All these issues should be taken into account as possible differentiation factors besides AD and addressed as potential sources of bias in the reported results. Moreover, small datasets and demographic variables mismatching in the population under study might lead to inconclusive results, since the model generalization would be unrealistic. In limitations related to the EEG experiment setup, [66] reported nonrecruitment of severe AD participants as they would not be able to undergo the experiment, consisting in resting-state EC during 3 minutes. In studies performing source localization, the most frequent limitation reported was the relatively low number of electrodes, which has a negative impact on the spatial resolution of the source localization results (Section 3.4.7).

Regarding the EEG processing, emphasis is often put on the manual selection of clean EEG epochs, which introduces human biases and cannot be reproduced. A limitation reported in a very recent study [80] is related to the resting-state EC condition: the dominance of alpha band in the spectral power, which is more marked in parietal and occipital electrodes. Indeed, a recent study has shown that the use of EEG epochs with lower alpha activity improves the discriminative power between AD patients and healthy controls [95].

Lastly, limitations related to the reported outcomes include the uncertainty of AD diagnosis using MMSE and other neuropsychological tests. Several studies measure the classification accuracy between AD or healthy controls using the results from these tests. However, neuropsychological tests do not provide 100% sure diagnosis; they do not work well in all dementia stages, and as they have lower sensitivity, it is difficult to detect early stages of AD [9]. Therefore, fluctuations in diagnoses with MMSE or other neuropsychological tests can occur and be detrimental for further results based on those scores. Additionally, not all studies performed longitudinal follow-ups nor corroborated the data from healthy controls and MCI and AD participants, as some AD participants could be suffering from a different dementia [79] and some MCI participants will not develop AD (Section 3.2.3).  Table 31 presents the abovementioned limitations.

### 3.6. Recommendations

After the discussion in previous subsections, various aspects worth to be addressed in future resting-state EEG-based studies are presented in Table 32 in the form of simple recommendations.

Throughout this review, we found that several studies do not present a detailed characterization of the cohorts participating in the study. Variables such as age, gender, and education level have been demonstrated to be confounding factors in AD [145, 162]. As such, it is recommended to provide as much information as possible on the study participants, indicating whether or not there are statistically significant differences in demographic variables between groups. In the same sense, it is important to inquire and report the pharmacological regime of the study participants to discard it as a confounding factor. From articles reporting limitations, a common issue is the possible mislabeling of participants (MCI, AD, and N) due to the methods used to diagnose the participants. In order to address this issue, studies must provide a clear description of the criteria used for participant inclusion and exclusion. Moreover, in the studies where the MCI cohort is considered as prodromal AD, it should be stated whether a follow-up was performed.

Regarding the study setup, a frequent issue that arises from this systematic review is the huge amount of different experimental setups that have been reported across the reviewed articles. While all the databases utilized by the studies included in this review used the same resting-awake eyes-closed protocol, the recording duration was extremely variable (Section 3.3.5), as well as the amount of EEG data utilized (Section 3.4.6). Experiment duration is a very important aspect to consider when designing the experimental setup, since MCI and AD participants are not always able to comply with the experiments, as reported by [66]. Regarding the EEG signal acquisition, most of the studies reported the use of the 10-20 international system as electrode placement guide. However, this information alone is not enough; the studies should provide a complete list of the actually used electrodes.

One direct consequence of this experimental variability is that most of the reviewed studies performed their analysis just on one dataset. While testing the efficiency of the developed methods on other datasets is highly advisable to verify if the results are realistic and can be generalized, this variability makes that practically impossible. As such, it is recommended that a standardization effort on EEG data collection and experimental protocol be put in place to facilitate cross-site, cross-country, and cross-database validation.

For EEG processing, in turn, the most used artifact-handling approach was the meticulous visual inspection by expert clinicians, which is inherently irreproducible and prone to errors. Consequently, even when EEG data is collected in the same conditions, the manual rejection of artifacts hinders the comparisons among different approaches of the same experimental setup. Studies could make use of AAR methods to report their results with manual selected EEG signals and contrast them to the ones obtained with automatically cleaned signals, as was done in [84].

Moreover, when recording EEG signals, less than half of the studies use EOG and very few use ECG electrodes. Registering eye and heart movements can help with artifact removal and thus should become standard during data recording. In addition, a clear description of the EEG signal epoching process should be provided and aspects like epoch length, epoch overlap, and number of epochs used need to be mentioned in the article. When source localization is performed, higher-density montages are desirable (≥25 electrodes) [182]. However, the participant comfort level needs to be taken into account, as it can be a source of bias in the study. Articles proposing innovative features should also test more well-established features that can be used as a baseline to be benchmarked against. The PSD-based features are a good candidate for baseline as they have been exhaustively studied and are easy to implement. For discriminative studies, articles should clearly detail the feature selection, cross-validation, and classification methods, because sharing data among these processes might lead to overfitting and optimistic biases. In a similar fashion, when multiple comparisons are reported, the statistical analysis subsection should report the post hoc correction method used for avoiding false positives.

Lastly, not every reviewed paper mentioned the limitations found during the study, this could be enlightening for the design of future studies. A solution for some of the reported limitations (Section 3.5.3) could be joining efforts to have free, publicly available EEG datasets. In this way, attempts should be made to create open-access EEG databases for the research community, where researchers can verify and test signal enhancement methods, proposed features, classification algorithms, and so on. An example of a successful initiative in a related field was the case of EEG-based brain-computer interfaces (BCI): having publicly accessible datasets has given great impulse to BCI research worldwide; such enterprise has been motivated by “The Future of Brain/Neural Computer Interaction: Horizon 2020” (BNCI Horizon 2020) project [190].

## 4. Conclusions

In this systematic review, a total of 112 journal articles published between January 2010 and February 2018 on the utilization of EEG for AD diagnosis and progression assessment were surveyed. In these papers, the most often reported goal was to discriminate between healthy controls and AD participants (59 articles). From these articles, crucial aspects were grouped under five main categories: study rationale, study population, experiment setup, EEG processing, and reported outcomes. Such aspects were reviewed, compared, and discussed, with the final goal of providing an overview of the state of the art on resting EEG for AD diagnosis and assessment.

In this review, limitations reported in the reviewed articles were also collected and discussed, with the aim of having an idea of the issues that need more attention in order to advance the use of EEG in AD research. Among these reported limitations, the limited number of datasets available to researchers appeared to be the most common one. Ultimately, it is hoped that this review will boost the research of EEG as a noninvasive, less-expensive, and potentially portable technique for AD study, assessment, and diagnosis, particularly for low- and middle-income countries which lack access to costly neuroimaging equipment.

## Figures and Tables

**Table 1:** Eligibility criteria.

*Inclusion*
Studies using EEG to assess AD progression
Studies using EEG to AD diagnosis
Studies using EEG to perform differential diagnosis between AD and other dementias

*Exclusion*
Studies on AD-related epilepsy
Studies without resting-state EEG recordings
Studies focused on dementias other than AD
Studies focused on the effects of AD treatment drugs
Studies on animals (nonhuman studies)
Studies not treating MCI as a prodromal stage for AD
Review articles

**Table 2:** Extracted items from each article.

Category	Data item	Description
Study rationale	Study goal	Application or aim of the article
	Other dementias	Differential diagnosis of different types of dementias with respect to AD

Study population	Sample size	Size of the population in the study
	Group matching	Groups matched (or not) by sample size, age, gender, and education level
	Following of MCI participants	Follow-up of MCI participants, when required

Experiment setup	Other modalities	Other modalities utilized beside EEG
	Number of electrodes and layout	Electrode number and positioning system
	External channels	Report the acquisition (or not) of EOG and ECG signals
	Resting-state recording state	EEG recorded only in resting state or with task performing too
	Experiment duration	Session duration of each experiment

EEG processing	Preprocessing	Survey on preprocessing techniques
	EEG bandwidth	Bandpass filtering of EEG signal and type of filters used
	Artifact handling	Artifact rejection and/or correction methods
	Effective sampling frequency	Sampling frequency of EEG data for feature extraction
	EEG epoching	Epoching process, length, and quantity of epochs
	Effective EEG signal duration	Length of EEG signal used for feature extraction
	Source localization	Survey on source localization methods when required
	EEG feature types	Survey on the types of EEG features used

Reported outcomes	Discriminative studies	Methods for discriminative task and reported results
	Assessment studies	Methods for assessment task and reported results
	Reported limitations	Limitations reported in the study

**Table 3:** Study goal description.

Study type	Study goal	Articles
Diagnosis (72)	AD vs Nold (48)	[58, 77–123]
	MCI vs AD (2)	[124, 125]
	MCI vs Nold (4)	[126–129]
	AD vs Nold vs others (6)	[37–41, 130]
	AD vs MCI vs Nold (12)	[131–142]

Progression assessment (18)	AD (3)	[143–145]
	AD vs Nold (1)	[146]
	MCI vs AD (3)	[147–149]
	MCI (11)	[150–160]

Diagnosis and progression assessment (22)	AD vs Nold (11)	[66, 161–170]
	AD vs MCI vs Nold (4)	[171–174]
	AD vs Nold vs others (2)	[26, 175, 176]
	AD vs others (1)	[42]
	MCI vs AD (3)	[177–179]

**Table 4:** Combination of AD diagnosis with other dementias.

Type of dementia	Articles
VaD	[26, 39, 40]
FtD/FTLD	[37, 175]
DLB	[41]
PDD	[38]
PDD/DLB	[176]
PDD/DLB/FtD	[42]
PDD/DLB/FtD/VaD	[130]

VaD: vascular dementia; FtD: frontotemporal dementia; FTLD: frontotemporal lobar degeneration; DLB: Lewy body dementia; PDD: Parkison's disease dementia.

**Table 5:** Datasets used repeatedly in the selected studies.

Datasets used in more than one study	Articles
22 subjects (11 AD, 11 Nold)	4 [80, 108–110]
24 subjects (10 mild AD, 14 Nold)	2 [91, 92]
27 subjects (20 probable AD, 7 Nold)	3 [104–106]
28 subjects (14 probable AD, 14 Nold)	3 [120–122]
34 subjects (22 probable AD, 12 Nold)	2 [112, 114]
34 subjects (17 AD, 17 Nold)	3 [77, 98, 111]
48 subjects (17 early AD, 16 MCI, 15 Nold)	4 [136–139]
62 subjects (3 databases: (a) 17 mAD, 24 Nold; (b) 5 mAD and 5 Nold; (c) 8 mAD and 3 Nold)	3 [78, 93, 101]
74 subjects (74 MCI)	9 [147, 152, 154, 156–160, 180]
79 subjects (79 probable AD)	4 [144–146, 162]
220 subjects (120 AD, 100 Nold)	2 [81, 115]

**Table 6:** Group matching according to the number of subjects, age, gender, and education.

Group matching	Articles
One group only (15)	[143–155, 158, 166]
Number, age, gender, education (8)	[26, 37, 66, 81, 103, 115, 124, 176]
Number, age, gender (10)	[38–40, 86, 89, 90, 102, 118, 119, 173]
Number, age, education (7)	[77, 95, 98, 99, 111, 164, 165]
Age, gender, education (4)	[82, 128, 162, 168]
Number, age (18)	[42, 80, 94, 100, 108–110, 112, 120–122, 132, 134, 136–139, 172]
Number, gender (2)	[88, 142]
Number, education (3)	[58, 79, 167]
Age, gender (3)	[78, 84, 123]
Age, education (3)	[129, 169, 171]
Number (8)	[83, 87, 97, 107, 117, 135, 140, 163]
Age (12)	[41, 91, 92, 96, 113, 114, 125–127, 141, 175, 178]
Not paired or no information (19)	[85, 93, 101, 104–106, 116, 130, 131, 133, 156, 157, 159–161, 170, 174, 177, 179]

**Table 7:** Combination of EEG with other modalities.

Modality	Biomarkers	Articles
MRI (11)	Cortical thickness, hippocampal atrophy, and other cortical density alterations	[41, 81, 147, 152–155, 166, 169, 171, 174]
MRI and SPECT (5)	Regional blood perfusion and other cortical density alterations	[156–160]
SPECT (1)	Anomalous activities of cerebral neurons in NAT (neuronal activity topography)	[179]
MRI and genetic (1)	Comparison of Genetic (ApoE) and neuroimaging alterations	[150]
Genetic data (1)	ApoE genotype; PSEN1 E280A mutation	[128]
PET (1)	Disease processes revealed by cortical hypometabolism	[82]

MRI: magnetic resonance imaging; SPECT: single-photon emission computed tomography; ApoE: apolipoprotein E; PET: positron emission tomography.

**Table 8:** Number of electrodes used by each selected study.

Electrode *N*	Articles
1–16 (14)	[85, 86, 88, 91, 92, 96, 100, 102, 108, 120–122, 142, 165]
17–32 (89)	[26, 37–42, 58, 78–84, 87, 89, 90, 93–95, 97, 101, 103–107, 109, 110, 112–119, 123, 125–127, 129–141, 143–164, 166–170, 173–179]
33–64 (2)	[66, 172]
65–128 (5)	[77, 98, 99, 111, 171]
129–256 (2)	[124, 128]

**Table 9:** Recording conditions.

Condition	Articles
Resting-awake EC (85)	[37, 38, 41, 42, 58, 66, 77–84, 86, 87, 90, 93–96, 98–115, 117–122, 124, 125, 127–130, 132, 134, 135, 140, 141, 146–149, 151–161, 164–169, 172–179]
Resting-awake EC + EO (13)	[39, 40, 85, 88, 89, 91, 92, 123, 126, 131, 133, 143, 163]
Resting-awake EC + EO + sensory stimulus (3)	[170, 171] visual stimulus, [150] auditory stimulation
Resting-awake EC + EO + cognitive tasks (8)	[144, 145, 162] episodic memory tasks, [136–139] backwards counting while finger tapping, [142] working memory task
Resting awake, eye condition not reported (3)	[26, 97, 116]

**Table 10:** Signal duration.

Description	Articles
*<*5 min (10)	[41, 66, 77, 98, 99, 111, 130, 146, 170, 175]
5–9 min (39)	[40, 80–82, 84, 85, 93, 101, 103, 115, 127, 134–136, 138, 141, 142, 145, 147–150, 152–160, 162, 163, 166, 169, 172, 176, 177, 179]
10–20 min (17)	[37, 86, 88, 89, 92, 109, 110, 120–124, 128, 131, 143, 164, 174]
*>*20 min (16)	[26, 38, 39, 91, 94, 107, 112–114, 119, 129, 137, 139, 144, 151, 178]
Not informed (30)	[42, 58, 78, 79, 83, 87, 90, 95–97, 100, 102, 104–106, 108, 116–118, 125, 126, 132, 133, 140, 161, 165, 167, 168, 171, 173]

**Table 11:** Filters.

Filter/preprocessing	Articles
Notch filter for power grid interference (35)	[38, 42, 58, 78, 84, 90, 92, 95, 100, 111–113, 119, 123, 127, 129, 136–141, 144–146, 148, 162, 164, 167, 170, 172, 174, 177]
Resampling (12)	[39, 40, 78, 83, 93, 96, 101, 128, 131, 138, 146, 177]
Rereference to common average (28)	[56, 77, 81, 82, 98, 99, 103, 111, 115, 124, 130, 147, 151–154, 158–160, 166, 167, 171, 173, 176–178, 180]
Interpolation of bad channels (3)	[77, 124, 128]

**Table 12:** Different upper limit bandwidths used by the selected EEG studies.

Upper limit (Hz)	Articles
≤25 (4)	[117, 125, 175, 179]
26–50 (57)	[37–40, 66, 77–83, 85–87, 89, 92, 94, 96, 98, 99, 101, 102, 104–106, 108–115, 120–123, 126, 127, 130–132, 134–136, 141, 143, 148, 149, 151, 163, 165, 167, 169, 171, 176]
51–75 (36)	[26, 42, 91, 103, 107, 118, 119, 124, 128, 133, 140, 142, 144–147, 150, 152–162, 164, 166, 168, 170, 173, 174, 177, 178]
≥76 (8)	[58, 84, 90, 95, 100, 137–139]
Not reported (7)	[41, 88, 93, 97, 116, 129, 172]

**Table 13:** Different lower limit bandwidths used by the selected EEG studies.

Lower limit (Hz)	Articles
≤0.5 (32)	[37, 42, 83, 84, 87, 103–106, 130, 131, 133, 141, 144–147, 149, 150, 153–160, 166, 169, 170, 173, 174]
0.5—*<*1 (36)	[26, 39, 40, 81, 82, 85, 86, 89, 94, 95, 100–102, 107–110, 115, 117–122, 124, 128, 129, 132, 135, 143, 148, 151, 163, 165, 167, 177]
≥1 (26)	[66, 77–80, 91, 92, 96, 98, 99, 111–114, 123, 125–127, 134, 140, 152, 162, 164, 171, 176, 179]
Not reported (18)	[38, 41, 58, 88, 90, 93, 97, 116, 136–139, 142, 161, 168, 172, 175, 178]

**Table 14:** Filter type.

Filter	Articles
FIR (26)	[39, 40, 42, 77, 80, 85, 86, 95, 96, 102, 108–111, 118–122, 127, 131, 140, 141, 145, 169, 171]
HOLS (1)	[124]
IIR (19)	[58, 78, 84, 90, 101, 112–114, 123, 125, 134–139, 172]
Not reported (68)	[26, 37, 38, 41, 66, 79, 81–83, 87–89, 91–94, 97–100, 103–107, 115–117, 126, 128–130, 132, 133, 142–144, 146–168, 170, 173–179]

**Table 15:** Artifact removal techniques.

Category	Method	Articles
Manual (65)	Epoch selection	[37, 58, 66, 77–80, 83, 86, 89–92, 94–96, 98, 99, 104–106, 108–111, 113, 114, 119–122, 125–127, 134, 135, 140, 141, 143, 147, 149, 151–161, 163–168, 170–175, 179]
Semiautomated (8)	ICA	[40, 107, 131, 169]
	ICA (IWASOBI)	[101]
	ICA (JADE)	[178]
	ICA in a sample and then ICA templates used to automatic removal	[39]
	ICA and wavelet denoising	[100]
Automated (19)	FASTER	[124, 128]
	Notch filter on blink frequency	[136–139]
	LR to EMG electrodes	[42, 81, 82, 103, 115, 144–146, 162, 176]
	wICA	[85, 132]
	BSS-SOBI-CCA and wICA	[84]
No filtering or no description (20)	—	[26, 38, 41, 87, 88, 93, 97, 102, 112, 116–118, 123, 129, 130, 133, 137, 142, 148, 150, 177]

BSS-SOBI-CCA: blind source separation based on second-order blind identification and canonical correlation analysis; ICA: independent component analysis; wICA: wavelet ICA; LR: linear regression; EMG: electromyographic.

**Table 16:** Sample frequency.

Frequency (Hz)	Articles
125 or 128 (22)	[37, 78, 81–83, 85, 91–93, 96, 101, 104–106, 115, 118, 126, 134, 135, 139, 176, 177]
200 or 256 (60)	[26, 38–40, 42, 58, 79, 80, 84, 87, 89, 90, 94, 95, 103, 107–110, 112–114, 116, 117, 125, 127, 129, 131–133, 136, 137, 140, 141, 143–147, 151–160, 162, 164–167, 169, 170, 173–175, 179]
500 or 512 (12)	[77, 98, 99, 111, 128, 138, 161, 163, 168, 171, 172, 178]
1000 or 1024 (11)	[41, 66, 86, 100, 102, 120–124, 149]
Not informed (7)	[88, 97, 119, 130, 142, 148, 150]

**Table 17:** Epoch duration.

Duration (s)	Articles
0.3–1 (8)	[77, 98, 99, 111, 129, 171, 177, 179]
1.1-2 (27)	[37, 39, 40, 66, 79, 81–83, 103, 115, 131, 141, 147, 152–160, 163, 169, 173, 174, 176]
2.1–5 (22)	[38, 42, 80, 89, 108–110, 124, 128, 140, 142–146, 148–150, 162, 166, 167, 175]
5.1–10 (21)	[58, 84–86, 90, 102, 104–106, 112–114, 120–122, 126, 151, 161, 165, 168, 170]
10.1–20 (8)	[78, 95, 101, 125, 127, 134, 135, 172]
21–70 (7)	[94, 96, 107, 118, 119, 132, 164]
Not informed (19)	[26, 41, 87, 88, 91–93, 97, 100, 116, 117, 123, 130, 133, 136–139, 178]

**Table 18:** Number of epochs.

Number of epochs	Articles
1–3 (12)	[78, 94–96, 107, 126, 127, 132, 134, 135, 164, 170]
4–10 (14)	[42, 86, 102, 118–122, 142, 151, 161, 165, 168, 173]
11–50 (20)	[37, 58, 66, 79, 80, 89, 90, 108–110, 113, 114, 124, 128, 140, 143, 146, 163, 167, 175]
51–150 (20)	[82, 99, 103, 131, 133, 141, 147, 152–160, 162, 166, 172, 174]
151–500 (7)	[39, 40, 98, 145, 171, 177, 179]
Not informed (39)	[26, 38, 41, 77, 81, 83–85, 87, 88, 91–93, 97, 100, 101, 104–106, 111, 112, 115–117, 123, 125, 129, 130, 136–139, 144, 148–150, 169, 176, 178]

**Table 19:** Effective EEG duration.

EEG duration (s)	Articles
8–30 (12)	[42, 66, 78, 126, 127, 132, 134, 135, 142, 170, 173, 175]
31–70 (20)	[37, 79, 86, 89, 94–96, 102, 107, 121, 122, 124, 143, 151, 161, 163, 165, 167, 168, 177]
71–150 (9)	[80, 99, 103, 108–110, 120, 137, 164]
151–300 (20)	[82, 98, 128, 131, 140, 141, 146, 147, 152–160, 171, 174, 179]
301–600 (9)	[40, 58, 90, 113, 114, 118, 119, 162, 166]
601–1500 (3)	[39, 145, 172]
Not informed (39)	[26, 38, 41, 77, 81, 83–85, 87, 88, 91–93, 97, 100, 101, 104–106, 111, 112, 115–117, 123, 125, 129, 130, 133, 136, 138, 139, 144, 148–150, 169, 176, 178]

**Table 20:** Slowing features.

Category	Description	Articles
Current source density	Source localization solutions	[26, 37, 66, 77, 82, 103, 115, 141, 150, 163, 176]

Spectral	Barlow's metrics	[178]
	Individual alpha peak (IAP)	[81–83, 103, 129, 137, 140, 151, 171, 175, 176, 178]
	Individual alpha3 alpha2	[147, 152, 153, 155–158, 160, 166]
	Individual beta peak	[178]
	PSD (absolute and relative band power)	[41, 42, 66, 81, 82, 84, 85, 88, 89, 92, 94–96, 98, 100, 102, 103, 107, 117, 121–124, 128–130, 135, 137, 140, 142–144, 149, 151, 154, 159, 161, 162, 167, 173, 174, 176, 178]
	PSD (band power ratios)	[41, 42, 88, 107, 129, 130, 137, 147, 152]
	PSD (central frequency)	[178]
	PSD (frequency peak in bands)	[79, 112–114]
	PSD (mean frequency in bads)	[42, 123, 140, 167]
	PSD (median frequency in bands)	[96, 137]
	PSD (modelling parameters)	[39, 40, 118]
	Wackermann's metrics	[178]

Spectrotemporal	Wavelet (continuous) parameters	[92]
	Wavelet (continuous) sparsification	[125]
	Wavelet (discrete) parameters	[38, 91]
	Wavelet maximum frequency	[178]

**Table 21:** Complexity features.

Category	Description	Articles
Entropy	Auto mutual information	[42, 140, 144, 146, 162]
	Epoch-based entropy	[93]
	Fuzzy entropy	[110, 140]
	Multiscale entropy	[146, 170]
	Multivariate multiscale entropy	[80]
	Quadratic sample entropy	[108]
	Sample entropy	[92, 96, 137, 140]
	Shannon entropy	[93, 146, 162]
	Spectral entropy	[100, 140, 146]
	Tsallis entropy	[146, 162]
	Wavelet entropy	[92]

Other	Bispectrum analysis	[122]
	Central tendency measure	[140]
	Correlation dimension	[93]
	Distance-based LempelZiv complexity (dLZC)	[109]
	Hjorth activity, mobility, and complexity	[137, 178]
	Lempel-Ziv complexity	[102, 137, 140]
	Visibility graphs	[126]
	Wavelet compression coefficients	[132]

**Table 22:** Synchronization features.

Group	Description	Articles
Directed model based	Direct transfer function	[83, 127, 134, 135]
	Direct directed transfer function	[127, 134, 135]
	Full frequency transfer function	[83, 127, 134, 135]
	Granger causality	[42, 127, 134, 135, 145, 162]
	Kullback–Leibler divergence	[127]
	Lateral asymmetry index (LAI)	[66]
	Phase slope index (PSI)	[96]
	Sugihara causality	[139]

Directed model free	Relative wavelet entropy	[172]
	Peak interregional transfer entropy delays (PITED)	[138]

Nondirected model based	Coherence	[41, 42, 78, 79, 83–85, 89, 96, 104, 105, 112, 120, 121, 127, 130, 134–136, 143–145, 149, 157, 158, 162]
	Coherence (wavelet)	[38, 105, 106, 119]
	Correlation	[78, 111, 127, 135]
	Correlation (amplitude envelopes)	[178]
	Detrended cross-correlation analysis (DCCA)	[86]
	Global field synchronization (GFS)	[127, 165]
	Global phase synchronization	[98]
	Global synchronization index	[164]
	Lagged linear connectivity (LLC)	[81, 115, 131, 141, 163, 169, 171]
	Multivariate phase synchronization (MPS)	[98]
	Omega complexity	[127, 134, 135]
	Phase lag index (PLI)	[124, 168]
	Phase synchrony	[78, 127, 134]
	S-estimator	[99, 127]
	Stochastic event synchrony	[127]

Nondirected model free	Coherence entropy coefficient	[127]
	Correlation entropy coefficient	[127]
	Mutual information	[42, 119, 127, 145]
	Permutation disalignment index	[148, 149]
	Synchronization likelihood	[97]
	Wavelet entropy coefficient	[127]

Others	Canonical correlation	[145]
	Global field power (GFP)	[37, 124]
	Graph theory metrics	[97, 111, 120, 124, 131, 136, 141, 148, 168, 169, 172]
	Static canonical correlation	[162]

**Table 23:** Neromodulatory features.

Description	Articles
Amplitude envelope, spectral analysis	[58, 84, 85, 90]
Amplitude envelope, statistics	[178]

**Table 24:** Nonbiological features.

Description	Articles
ANN extracting spatial content from EEG	[133, 177]
Back-predictive model	[116]
Linear predictive model	[116]
Paraconsistent artificial neural network (PANN) using morphological analysis of EEG	[87]
Symmetric predictive model	[116]

**Table 25:** Classification, statistical analysis, or both.

Description	Articles
Statistical (35)	[26, 37, 39, 66, 78, 80, 82, 88, 90, 95, 99, 104, 106, 108, 111, 119, 120, 131, 132, 141, 142, 161–165, 167–170, 172–174, 179, 188]
Classification (36)	[40, 41, 58, 84, 85, 87, 91–94, 96, 97, 100, 101, 105, 112–117, 123–126, 129, 130, 133, 135–140, 171, 177]
Both (23)	[38, 42, 77, 79, 81, 83, 86, 89, 98, 102, 103, 107, 109, 110, 118, 121, 122, 127, 128, 134, 175, 176, 178]

**Table 26:** Statistical analysis strategy in the selected studies.

Description	Articles
ANOVA	[66, 81, 82, 90, 95, 99, 102–104, 106, 121, 122, 124, 131, 141, 142, 168–170, 173–176]
Anterior hub ratio	[172]
chi squared	[66, 161, 168]
Correlation	[107]
Correlation *P*	[82, 90, 141, 176]
Correlation *P* split half	[90]
Cost function	[119]
Graph analysis	[141]
Kruskal-Wallis	[90, 109, 110]
LDA	[38, 108]
Lilliefors test	[110]
Log-*F*-ratio (LORETA solutions)	[26, 37]
Mahalanobis D2	[167]
MANCOVA	[39]
Mann–Whitney	[42, 78, 80, 89, 127, 134, 164]
MANOVA	[172]
Mean and standard deviation	[120, 132]
PCA	[83]
Quadratic univariate regressions	[162]
SNK	[142]

LDA: linear discriminant analysis; MANCOVA: multivariate analysis of covariance; SNK: Student–Newman–Keuls.

**Table 27:** Feature selection.

Feature selection methods	Articles
AUC maximization	[58, 83, 112, 122]
BFE	[133]
Consistency-based filter (CBF), correlation-based feature selection (CFS), filtered subset evaluator (FSE), Chi squared (CS), gain ratio (GR), relief-*F*, symmetrical uncertainty (SU), and ensemble feature selection (EFS)	[114]
Correlation-based pursuit	[129]
FCBF	[140]
Fit-curve model	[40]
Genetic	[41, 130]
Logistic regression	[107, 178]
Manual	[96]
OFR	[135]
*p* value	[81, 109, 126, 127, 176]
PCA	[139]
Ranking by Fisher ratio score	[38]
Reverse sequential feature selection	[42]
SVD	[77]
SVM classifier (best performers)	[85, 136–138]

BFE: best feature extraction; FCBF: fast correlation-based filter; OFR: orthogonal forward regression; SVD: singular value decomposition.

**Table 28:** Cross validation methods.

Description	Articles
5-fold CV	[122]
10-fold CV	[38, 40, 41, 84, 85, 94, 130]
100-fold CV	[126]
500-fold CV	[115]
Dataset split in train and test set splits	[83, 94, 105, 112, 117, 123, 129, 133, 140, 177, 178]
LOSO	[42, 58, 77, 85, 91–94, 98, 100, 108, 109, 113, 114, 125, 127, 134–139, 171]
Leave one epoch out	[109]

CV: cross-validation; LOSO: leave one subject out.

**Table 29:** Classifying Strategy.

Classifier	Articles
ANN	[101, 115, 117, 123, 126, 133, 140, 177]
ANOVA	[38]
Autoregressive models	[116]
Back predictive model	[116]
Decision tree	[91, 92]
k-nearest neighbor	[129, 133]
LDA	[40, 86, 93, 98, 125, 127, 133–135, 140, 171]
LR	[107, 113, 124, 128, 133, 178]
LRA	[41]
Nave Bayes	[133]
PANN	[87]
Parzen classifier	[133]
PCA	[139]
PDM-based model	[96]
PNN	[105]
QDA	[127, 133, 140]
ROC	[83, 109, 124, 130, 175, 176]
SMO	[133]
SVM	[58, 77, 84, 85, 94, 97, 100, 102, 112–114, 133, 136–139]
Takagi-Sugeno neurofuzzy inference system	[129]

ANN: artificial neural network; LDA: linear discriminant analysis; LR: logistic regression; LRA: logistic regression analyses; PANN: paraconsistent artificial neural network; PDM: principal dynamic mode; PNN: probabilistic neural network; QDA: quadratic discriminant analysis; SMO: sequential minimal optimization.

**Table 30:** AD progression assessment.

Description	Articles
ANOVA	[26, 147, 153, 155–160]
ANCOVA	[152, 154]
ANOVA 2 way	[161]
Chi squared	[151]
Correlation	[144]
Correlation (Pearson)	[147, 148, 153, 155–160, 163, 165, 169, 172–176]
Correlation partial	[143]
Correlation (Spearman)	[66, 143, 164, 168]
Genetic search multiple markers	[178]
K-means	[165]
LDA	[171]
Linear regression	[42]
Mahalanobis D2	[167]
Mann–Whitney	[151]
Quadratic ordinary least squares regression models	[145]
R2	[42, 144–146, 162]
Scheffes test	[26]
*t*-test	[151]
*Z*-standardized statistic	[167]
Wilcoxon rank-sum test	[149]

ANCOVA: analysis of covariance.

**Table 31:** Reported limitations.

Category	Description	Articles
Population	Small number of subjects in the study	[37, 58, 80, 83, 86, 99, 101, 109–111, 121, 124, 128, 135, 139, 140, 153, 159, 163, 164]
	Merged databases are different due to local implementations	[81]
	Lack of different stages in AD cohort	[86, 121, 135]
	AD cohort includes participants taking antidementia drugs	[81, 83, 163, 164]
	Lack of population matching, age, gender, and/or education	[66, 90, 99, 135]
	Possible preclinical AD in N cohort	[81]
	Prodromal AD was applied in aMCI with A*β*42	[150]

EEG experiment setup	No severe AD as hard to perform EEG recordings	[66]
	Presence of dominant alpha activity during EC condition	[80]
	Differences in datasets due manual artifact handling	[58, 81, 163]
	Low number of electrodes for source localization methods	[37, 82, 163]
	Low number of electrodes for connectivity analysis	[83, 145]
	Low number electrodes for advanced AAR methods	[84]

Reported results	Lack of research for other dementia types	[39, 109, 110, 139, 163]
	Lack of longitudinal approach for N, MCI, AD populations	[66, 128, 138, 155, 160, 170]

**Table 32:** Recommendations.

Recommendations for future EEG-based AD studies
Provide detailed population characteristics
Describe how the AD diagnosis was performed
Mention whether the MCI participants were followed-up
Detail EEG experiment in duration and phases
Use standard EEG layouts
Mention not only the quantity of channels but their location
Define EEG processing in more detail
Use standard features such as PSD features as baseline
Describe artifact handling strategies
